# Subungual melanoma of the fifth digit: A case report of a rare cutaneous malignancy documented in Syria

**DOI:** 10.1097/MD.0000000000046997

**Published:** 2026-01-23

**Authors:** Osama Haj Osman, Yhia Alsayed, Sawsan Abo Saada, Ahmad Alkhamis, Nour Hintaieh, Lina Ghabreau, Aladdin Etr

**Affiliations:** a Department of Medical Research, Faculty of Medicine, Aleppo University, Aleppo, Syria; b Department of Plastic Surgery, Faculty of Medicine, Aleppo University, Aleppo, Syria.

**Keywords:** acral lentiginous melanoma, case report, dermatological malignancy, nail unit melanoma, neoplastic nail lesion, subungual melanoma

## Abstract

**Rationale:**

Subungual melanoma is the rarest subtype of cutaneous melanoma, accounting for approximately 1.9% of cases. Involvement of the fifth digit is exceptionally uncommon, often contributing to delayed diagnosis and poor outcomes.

**Patient concerns:**

We report a case involving a 45-year-old male patient who presented with longitudinal melanonychia, a positive Hutchinson’s sign, and ulceration of the nail plate on the right fifth finger.

**Diagnoses:**

Histopathological analysis confirmed subungual melanoma with a Breslow thickness of 4.3 mm.

**Interventions:**

The patient underwent surgical amputation at the proximal interphalangeal joint, accompanied by a sentinel lymph node biopsy. The excised specimen demonstrated clear margins, and the sentinel lymph node biopsy was negative. Contrast-enhanced computed tomography of the chest, abdomen, and pelvis revealed no evidence of metastatic disease.

**Outcomes:**

At 2-month follow-up, the patient reported good wound healing and no evidence of recurrence, though he was subsequently lost to follow-up. This case highlights the diagnostic challenges of subungual melanoma, particularly in the fifth digit, and underscores the importance of maintaining a high index of suspicion for pigmented nail lesions.

**Lessons:**

Early biopsy and timely surgical management remain critical for improving prognostic outcomes in rare presentations of melanoma.

## 1. Introduction

Cutaneous melanoma is a highly aggressive dermatologic malignancy arising from melanocytes, the pigment-producing cells of the skin, and is responsible for nearly 75% of all skin cancer-related deaths.^[1]^ It is classified into 4 major subtypes: nodular melanoma, lentigo maligna melanoma, superficial spreading melanoma, and the least common, acral lentiginous melanoma (ALM).^[2]^

Subungual melanoma (SUM) is a rare variant of ALM that originates in the nail matrix. It represents the rarest form of cutaneous melanoma, accounting for approximately 1.9% of cases.^[3]^ SUM occurs more frequently in the toenails than in the fingernails, with hand involvement comprising only 0.3% of all cutaneous melanomas according to the Melanoma Institute Australia database.^[4]^ Within the hand, the fifth digit is the least commonly affected site, underscoring the exceptional rarity of the present case.^[1]^

SUM typically arises in individuals between 50 and 70 years of age.^[2]^ Reported risk factors include family history, chronic inflammation, trauma, and mechanical stress.^[5]^ The infrequent involvement of the fifth digit may reflect its lower exposure to mechanical stress compared with other digits.

Published reports of SUM affecting the fifth finger remain scarce. Here, we describe the case of a man who underwent successful amputation of the right fifth finger following a diagnosis of subungual melanoma.

This work has been reported in line with CARE checklist-2013 criteria.

## 2. Case presentation

**Figure 1.**
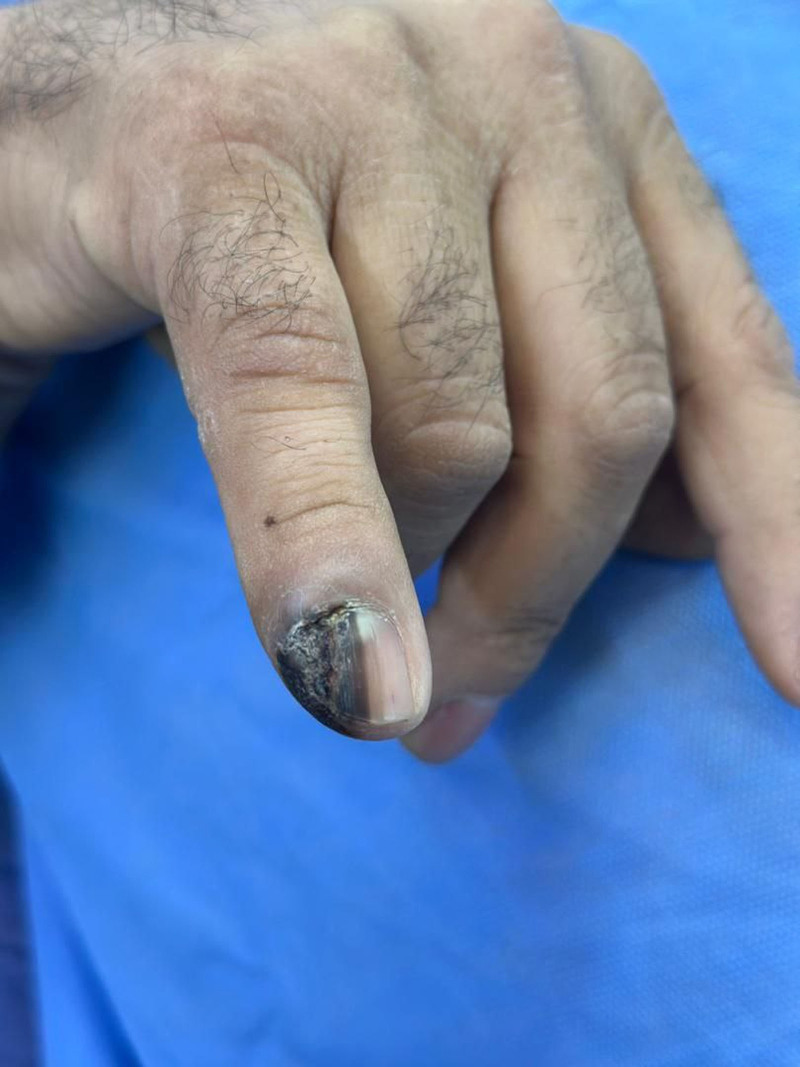
Fifth finger upon presentation. It exhibits a positive Hutchinson´s sign with an ulcerated lateral edge of the nail.

**Figure 2.**
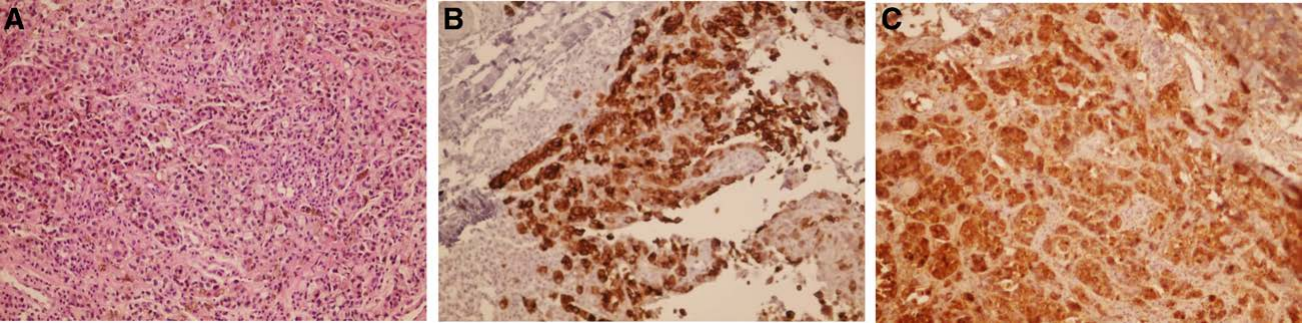
Dermal malignant proliferation of large round cells with large hyperchromatic pleomorphic nuclei and prominent nucleoli, eosinophilic cytoplasm, abundant melanin pigmentation, with few scattered atypical mitoses. (A) Hematoxylin and Eosin, (B) S100, (C) HMB45. Original magnification × 200.

**Figure 3.**
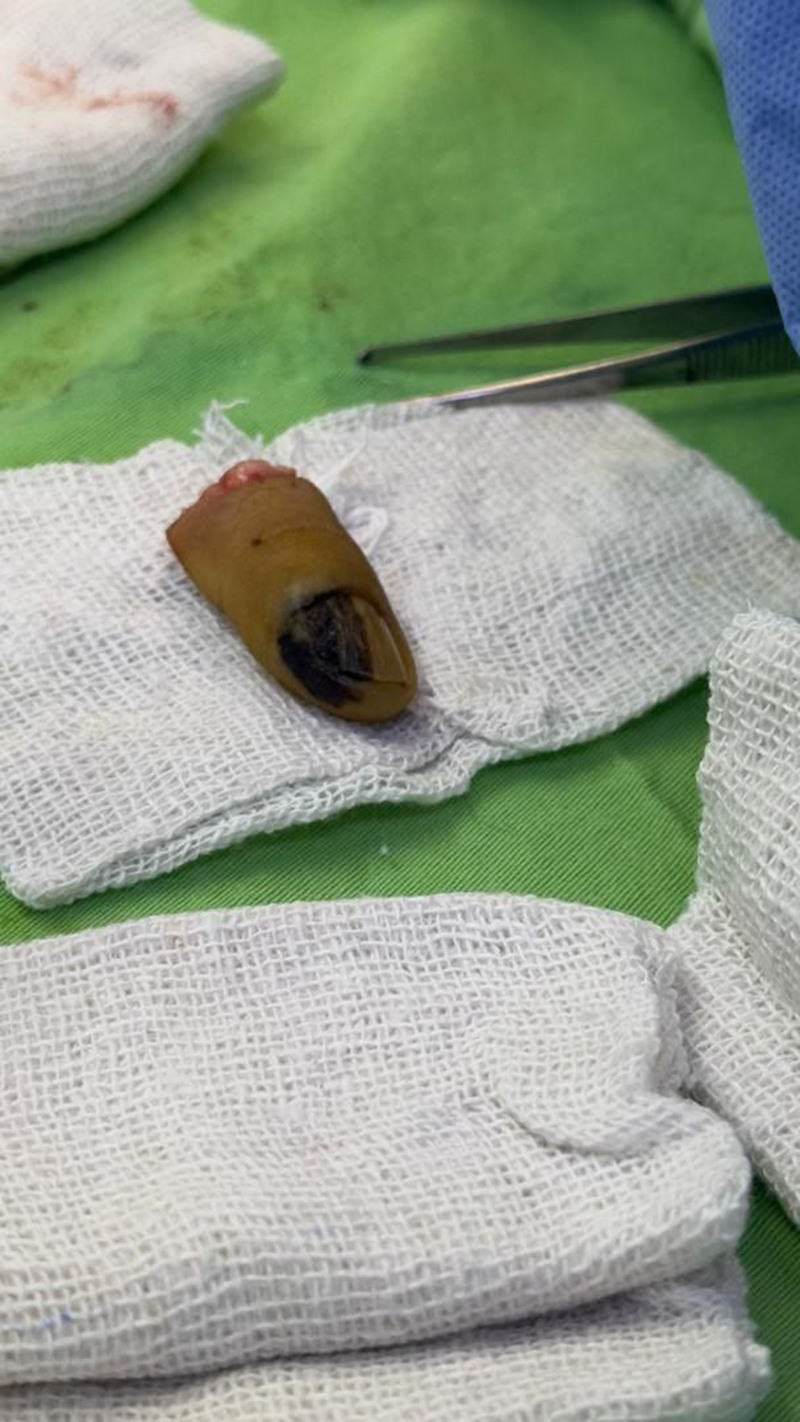
Intraoperative view of the affected finger after amputation.

A 45-year-old man presented to the dermatology department with a painless pigmented lesion on the nail of his right fifth finger (Fig. 1). The lesion had first appeared 18 months earlier and progressively increased in size. The patient reported no recent history of trauma or infection affecting that finger. His medical history was unremarkable, and he reported no allergies, medications, alcohol consumption, or tobacco use. Family history was notable for melanoma of the knee in his father. On clinical examination, the lesion was non-tender to palpation and demonstrated longitudinal melanonychia (LM) extending to the lateral and distal nail folds (Hutchinson sign), with ulceration along the lateral edge of the nail plate. A plain radiograph of the hand was unremarkable, and ultrasonography revealed no significant enlargement of the axillary lymph nodes. Laboratory investigations were within normal limits. Given the high suspicion of malignancy, an incisional biopsy was performed under local anesthesia and submitted for histopathological analysis. The biopsy specimen measured 0.6 × 0.2 × 0.4 cm and demonstrated malignant proliferation within the dermis, with positive immunohistochemical staining for S100 and HMB45. These findings were consistent with subungual melanoma (Fig. 2). Because the lesion was suspected to involve the distal interphalangeal joint, surgical excision of both the distal and intermediate phalanges was performed under general anesthesia, accompanied by sentinel lymph node biopsy of the axillary nodes. Intraoperatively, methylene blue was injected around the tumor site to map lymphatic drainage. Six blue-stained sentinel nodes were identified, dissected, and submitted for pathological examination. A reconstructive surgeon performed the procedure without complications, and the excised finger specimen was sent to the pathology department for analysis (Fig. 3). The specimen measured 4.9 × 1.1 × 1.6 cm and demonstrated clear surgical margins. The Breslow tumor thickness was 4.3 mm. Moreover, the sentinel lymph node biopsy was negative.

**Figure 4.**
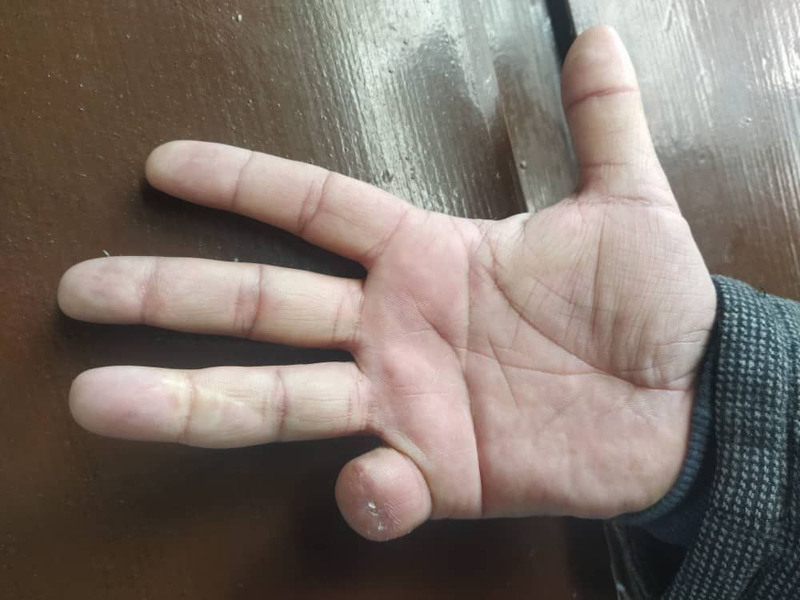
The right hand after 2 mo of follow-up.

**Figure 5.**
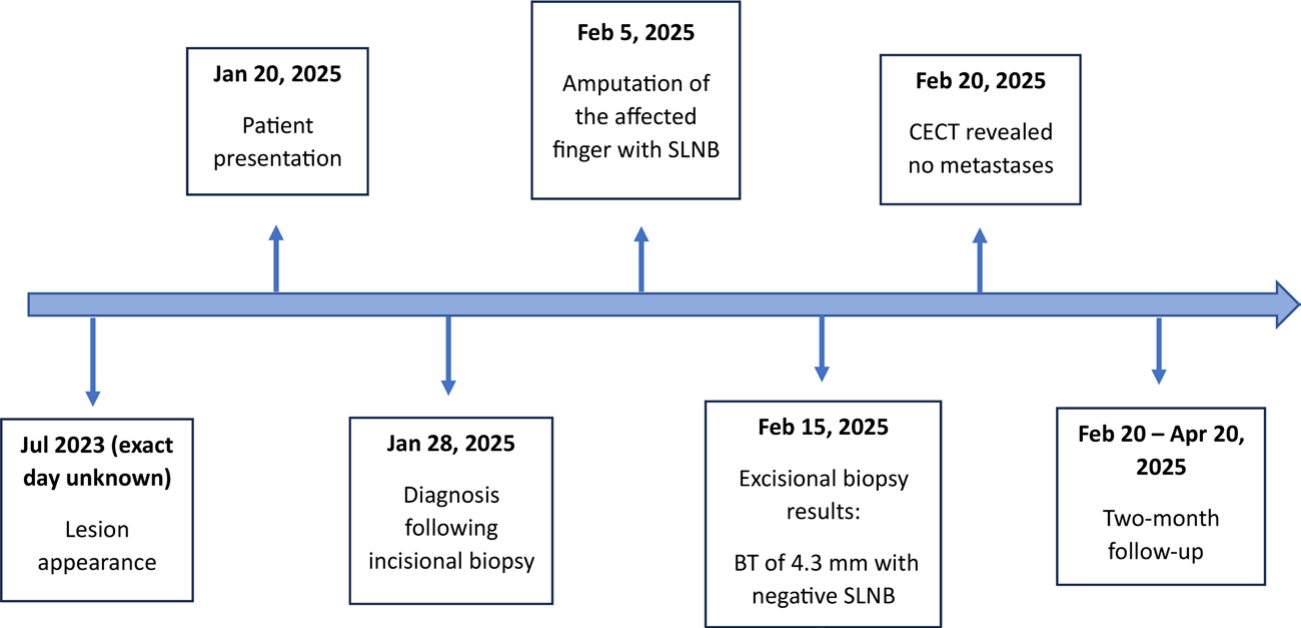
A brief timeline of the patient’s history with surgical management and follow-up period. BT = Breslow thickness, CECT = contrast-enhanced computed tomography, SLNB = sentinel lymph node biopsy.

Given the poor prognosis associated with this tumor thickness, contrast-enhanced computed tomography of the chest, abdomen, and pelvis was performed, revealing no evidence of metastatic disease. At the 2-month follow-up, the patient reported feeling well, with good healing of the surgical wound (Fig. 4). Unfortunately, follow-up could not be extended beyond 2 months, as the patient was lost to follow-up thereafter (Fig. 5).

## 3. Discussion

### 3.1. Epidemiology

Nail unit melanomas (NUMs) are generally believed to originate in the nail matrix, the primary site of melanocytes within the nail unit, where abnormal proliferation initiates tumor development.^[6]^ On rare occasions, NUMs may arise from the epithelium of the nail bed or the adjacent epidermis.^[7]^

Unlike most other melanoma subtypes, ALM is not associated with chronic sun exposure or ultraviolet (UV) radiation. Although the prevalence of ALM is relatively consistent across racial and ethnic groups, it accounts for only 1% to 2.5% of melanomas in white individuals, compared with 15% to 35% in those with darker skin and 50% to 58% in Asian populations. This disparity may reflect the greater susceptibility of lighter-skinned individuals to other melanoma subtypes linked to UV exposure. However, the literature provides limited insight into racial differences specifically related to NUM.^[8,9]^ Our patient had Fitzpatrick skin type III.

Subungual melanoma most commonly occurs in the nails of the thumb, hallux, and index finger. However, it has been documented in every digit, with the fifth finger representing the least frequent site of involvement.^[1,10]^

Interestingly, this case represents the third instance of subungual melanoma diagnosed at our hospital within the past 2 years. Notably, one of the previous cases also involved the fifth finger, while another was an amelanotic melanoma affecting the third finger. All 3 patients originated from a rural area in Aleppo Province, northern Syria.^[1,2]^

### 3.2. Genetics

Mutations in BRAF, NRAS, and KIT, as well as amplifications of CCND1, CDK4, MITF, and TERT, are well established in ALM. A comparative study of 54 NUM cases and 78 ALM cases without nail involvement demonstrated that KIT mutations were more frequent in NUM (16% vs 3% in ALM), while KRAS mutations were observed predominantly in NUM (5% vs 0% in ALM). In contrast, BRAF mutations were almost exclusively identified in ALM (22% vs 3% in NUM), and NRAS mutations were detected in 37% of ALM cases compared with only 9% of NUM cases. These findings underscore the molecular and genetic distinctions between the 2 melanoma subtypes.^[11]^

Although molecular profiling of KIT, KRAS, and BRAF mutations may provide clinically valuable insights, financial limitations precluded genetic testing in the present case.

### 3.3. Clinical evaluation

Approximately 2-thirds of subungual melanomas (SUMs) present clinically as LM, defined as a brown-to-black pigmented band running longitudinally along the nail plate.^[9]^ However, a variety of benign conditions may also produce LM, including fungal melanonychia, bacterial infections (such as those caused by *Pseudomonas aeruginosa*), exogenous pigment deposition, subungual hematoma, melanocytic tumors, drug-induced changes, and melanocytic processes such as benign melanocytic activation, lentigo, and nail unit nevi.^[12]^ Clinicians must therefore carefully distinguish between benign and malignant causes, maintaining a high index of suspicion for SUM.

Additional features concerning for SUM include heterogeneity of the pigmented band, progressive darkening or widening (exceeding 3 mm or more than 40% of the nail plate width), and a triangular configuration (wider proximally than distally). Other worrisome signs include purulence, bleeding, nail splitting, and Hutchinson sign, defined as extension of pigment onto the adjacent periungual skin.^[13,14]^ Moreover, up to 25% of nail melanomas are amelanotic, presenting as a pink, red, or flesh-colored papule, or as longitudinal erythronychia often accompanied by onycholysis, notching, splitting, bleeding, or ulceration.^[14,15]^

Our patient, with a familial predisposition to melanoma, presented with LM, a positive Hutchinson sign, and an ulcerated lateral nail margin, findings highly suggestive of malignant subungual melanoma.

### 3.4. Diagnostic methods

Dermoscopy is a valuable tool for differentiating subungual melanoma from benign melanocytic pigmented lesions. By using a dermatoscope, clinicians can visualize subsurface structures not apparent to the naked eye. Typical dermoscopic features of subungual melanoma presenting as LM include a brown-to-black background with longitudinal lines that exhibit irregularities in color, width, and spacing, often accompanied by loss of parallelism. Despite its utility in identifying clinical clues suggestive of malignancy, dermoscopy lacks sufficient sensitivity to detect all cases of subungual melanoma. Consequently, matrix biopsy followed by histopathologic examination remains the diagnostic gold standard^[9,16]^

In this case, dermoscopy was not performed prior to biopsy, and therefore no dermoscopic images are available for inclusion.

The choice of biopsy technique depends on the band’s position, whether lateral or medial, and the pigment’s location within the nail matrix, either proximal or distal. Options include nail clipping, punch biopsy, tangential shave technique, transverse matrix incisional biopsy, and lateral longitudinal biopsy.^[9]^

Given the lateral positioning of the lesion in our case, a lateral longitudinal biopsy was performed.

Immunohistochemical staining patterns are variable. An international survey conducted by the European Nail Society and the Council for Nail Disorders indicated that Melan-A/ MART-1 remains the preferred marker for identifying melanocytes within the nail unit.^[17]^

In our case, S100 and HMB45 stains were positive, while Melan-A was not available in our country.

Histopathologic features suggestive of subungual melanoma include lentiginous proliferation characterized by solitary melanocytes outnumbering cell nests, an increased density of junctional melanocytes, and prominent pagetoid spread. Additionally, these lesions often demonstrate marked cellular atypia, increased mitotic activity, and a notable junctional lymphocytic inflammatory response.^[18]^

Breslow thickness, which quantifies tumor depth from the granular layer of the epidermis to its deepest point, is a key parameter in TNM staging of cutaneous melanoma. However, due to the close anatomical relationship among the nail matrix, nail bed, and underlying bone, its reliability is reduced when applied to subungual melanoma.^[9,16]^ In our patient, the Breslow thickness was 4.3 mm, indicating a poor prognosis.

Diagnosis of subungual melanoma is frequently delayed. Contributing factors include the absence of a systematic clinical approach to pigmented nail lesions, wide variation in clinical presentation (including a high incidence of amelanotic melanoma), lack of visible nail changes during the radial growth phase, inadequately performed biopsies, and errors in histopathologic interpretation.^[9]^ In our case, there was an 18-month delay in diagnosis, as the patient refused to seek medical assessment during that period.

### 3.5. Management

For the management of subungual melanoma (SUM) in situ, surgical options may include Mohs micrographic surgery, which has better outcomes when MART-1 staining is employed. More commonly, wide local excision is recommended, requiring complete removal of the nail unit, including the nail plate, nail bed, and nail matrix. The resulting defect can be repaired with either a split-thickness or a full-thickness skin graft, or it may be allowed to heal by secondary intention. In cases of invasive SUM, such as in our patient, amputation at the level of the most distal unaffected interphalangeal joint is recommended.^[16]^

Clinical evaluation of lymph node involvement is essential for staging in both melanoma in situ and invasive melanoma. Sentinel lymph node biopsy is recommended for lesions thicker than 1 mm, or for lesions under 1 mm with a mitotic rate exceeding 1/mm², as these features indicate a more aggressive course. If sentinel lymph node biopsy is positive, a regional lymph node dissection should follow.^[16]^

In our case, the lesion was invasive melanoma with a thickness exceeding 4 mm; therefore, sentinel lymph node biopsy was performed and returned negative results.

Beyond surgical excision, patients with advanced NUM may be considered for emerging targeted and immunotherapeutic approaches. Therapies inhibiting BRAF and MEK, such as vemurafenib and trametinib, represent potential treatment options for NUM. Additionally, high-dose interferon alfa-2b (HDI) is FDA-approved for adjuvant therapy in resected high-risk melanoma. Immunotherapy may involve antibodies targeting cytotoxic T-lymphocyte-associated antigen 4 (CTLA-4), such as ipilimumab and tremelimumab.^[9]^

Our patient expressed complete satisfaction with the management received and personally thanked the hospital staff for their care and support. Unfortunately, follow-up could not be extended beyond 2 months, as the patient was lost to follow-up thereafter.

## 4. Conclusion

Subungual melanoma represents the rarest subtype of cutaneous melanoma, and involvement of the fifth digit is exceptionally uncommon, thereby presenting a significant diagnostic challenge. Clinicians should maintain a high index of suspicion when assessing nail lesions, particularly those characterized by LM or subtle features suggestive of an amelanotic variant. Targeted institutional efforts to enhance clinician and public awareness of nail-associated dermatologic malignancies are essential to minimize diagnostic delays, as illustrated by this case. Such initiatives may contribute to earlier detection, thinner tumors, and improved prognostic outcomes, with particular emphasis on rural and underserved regions where access to specialized medical services remains constrained.

## Author contributions

**Conceptualization:** Osama Haj Osman, Yhia Alsayed, Sawsan Abo Saada, Ahmad Alkhamis.

**Data curation:** Osama Haj Osman.

**Investigation:** Osama Haj Osman, Yhia Alsayed, Sawsan Abo Saada, Ahmad Alkhamis.

**Resources:** Nour Hintaieh, Lina Ghabreau, Aladdin Etr.

**Supervision:** Lina Ghabreau, Aladdin Etr.

**Validation:** Lina Ghabreau, Aladdin Etr.

**Writing – original draft:** Osama Haj Osman, Yhia Alsayed, Sawsan Abo Saada, Ahmad Alkhamis.

**Writing – review & editing:** Osama Haj Osman.
